# Supplementary material for: E-Cigarette Aerosol Exposure Favors the Growth and Colonization of Oral Streptococcus mutans Compared to Commensal Streptococci
Source: Microbiol Spectr. 2022 Apr 4;10(2):e02421-21. doi: 10.1128/spectrum.02421-21 (PMC9045065; doi:10.1128/spectrum.02421-21)
Supplement: SUPPLEMENTAL FILE 1 — Supplemental material. Download SPECTRUM02421-21_Supp_1_seq9.pdf, PDF file, 0.8 MB [file spectrum02421-21_supp_1_seq9.pdf]

Supplemental Materials

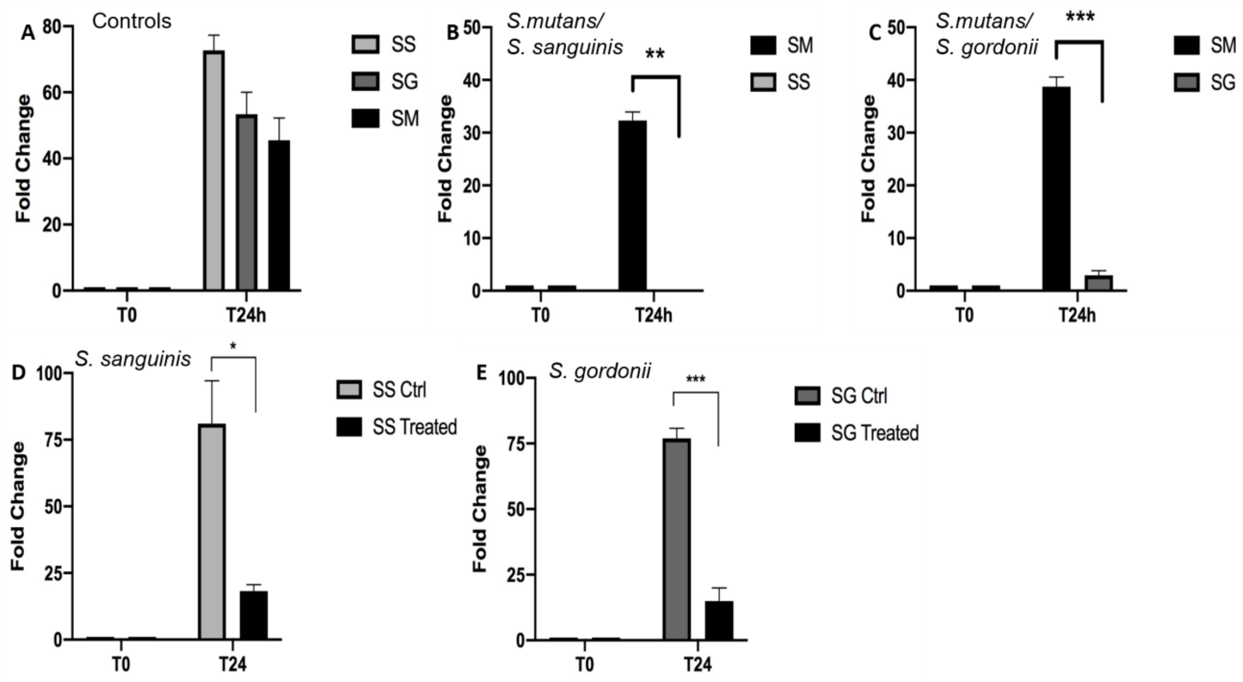

S1 Fig. *S. mutans* outcompetes *S. sanguinis* and *S. gordonii*.
(A) Overnight culture of all three strains were diluted 1:10 and grown for 24 hours in fresh TSB medium. CFUs/ml were counted at time 0 (T0) and 24 hours later (T24) and fold change calculated. (B) *S. mutans* and *S. sanguinis* were diluted 1:10 after overnight culture and mixed in equal volume together for 24 hour growth in fresh TSB. CFU/mL of each species were counted at time 0 and time 24 hours and fold change calculated. (C) Overnight cultures of *S. mutans* and *S. gordonii* were diluted 1:10, mixed in equal volume together and grown for 24 hours in fresh TSB, and CFU/mL of each species were counted at time 0 and time 24 hours. (D-E) Overnight cultures of *S. sanguinis* and *S. gordonii* grown in TSB media supplemented with *S. mutans* supernatant. CFUs/ml were counted at time 0 (T0) and 24 hours later (T24) and fold change calculated. The data represent mean ± standard error of the mean (SEM; n = 3) of three biological replicates. The experiment was repeated at least twice. Groups were compared to control using Student T-test (Welch's correction). $p<0.05$*; $p<0.01$**; $p<0.001$***

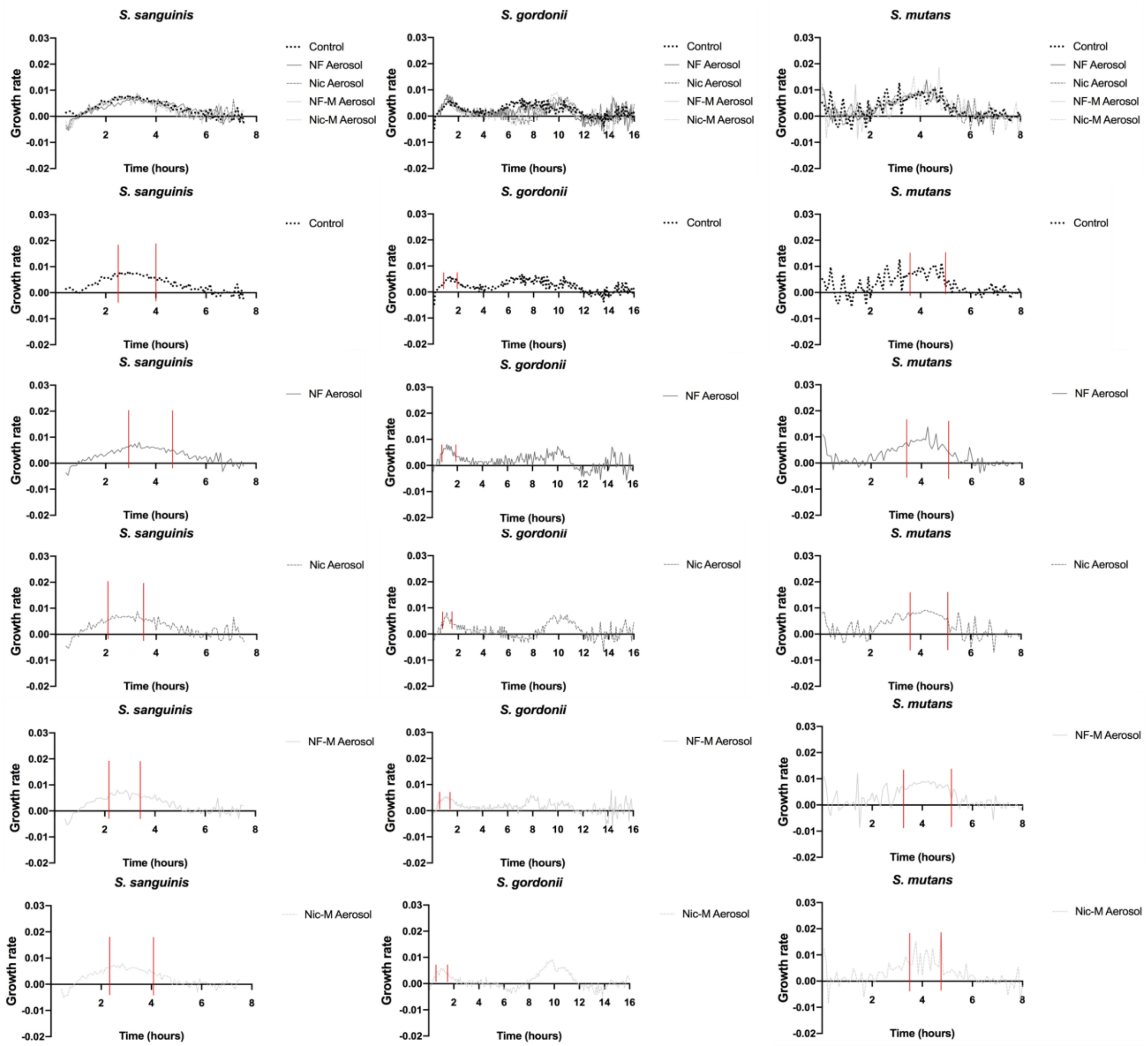

S2 Fig. Derivative graphs for aerosol growth curves.
Taking the derivative of the Log_2 transform of the growth curves shown in Fig 1 and 2 served to find the fastest rate of change occurring in the exponential phase of growth for each condition. The fastest rate of change aligns with the highest peak in each derivative graph. The times of these peaks were plotted to obtain the linear regression slopes shown in Fig 1 and 2.

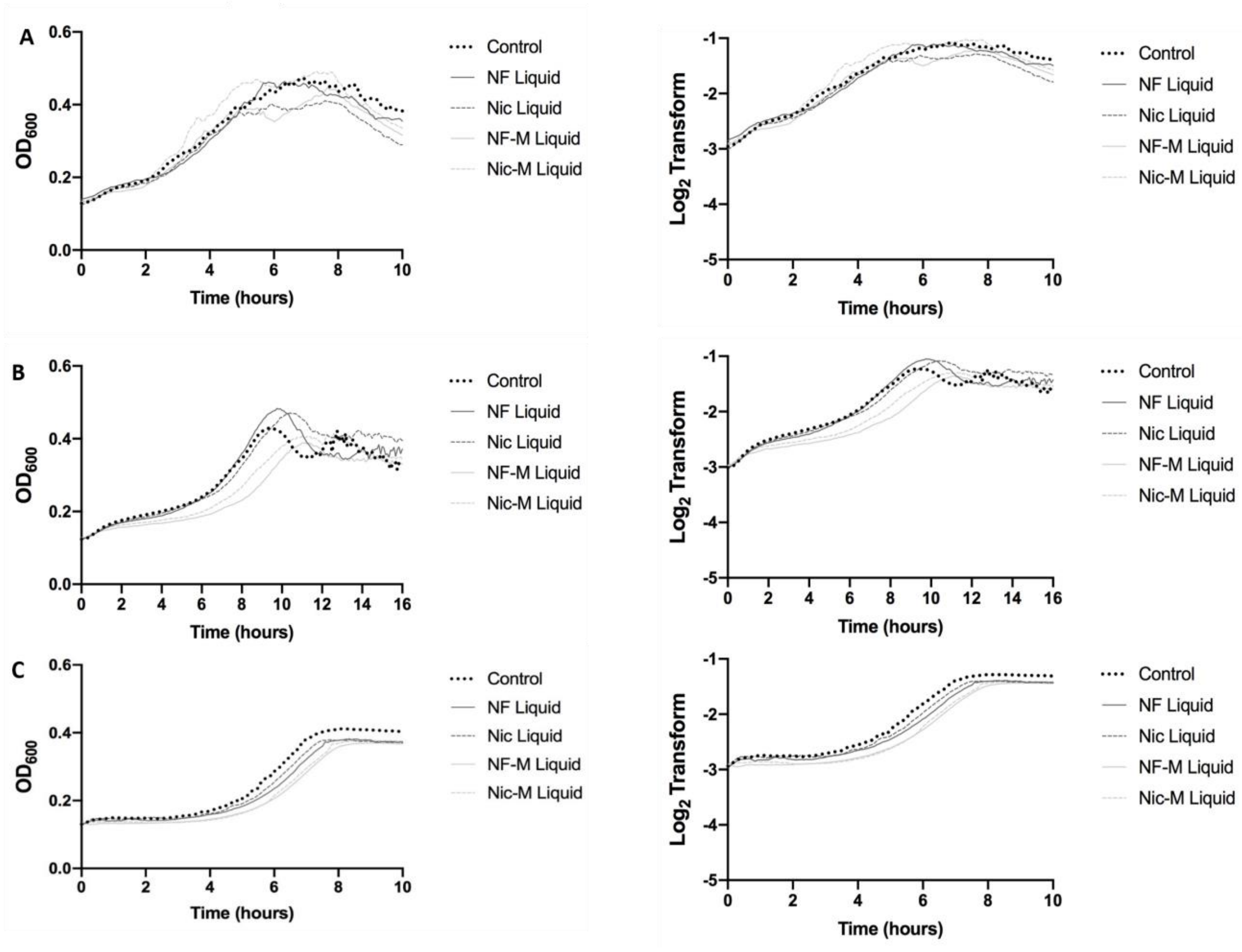

S3 Fig. E-liquids minimally affect the growth of *S. sanguinis* and *S. gordonii,* but not of *S. mutans*. E-liquid did not affect any of these bacteria biofilm formation capacity.
For growth curves, overnight *S. sanguinis* (A) and *S. gordonii* (B), and *S. mutans* (C) cultures were diluted 1:10 into 1% e-liquid media and were grown 24 hours, measuring optical density at 600 nm (OD_{600}) every 5 minutes. Average values were plotted as OD_{600} (left panels) and Log_2 transform (right panels). Data are representative of one biological replicate (n=3) repeated at least twice.

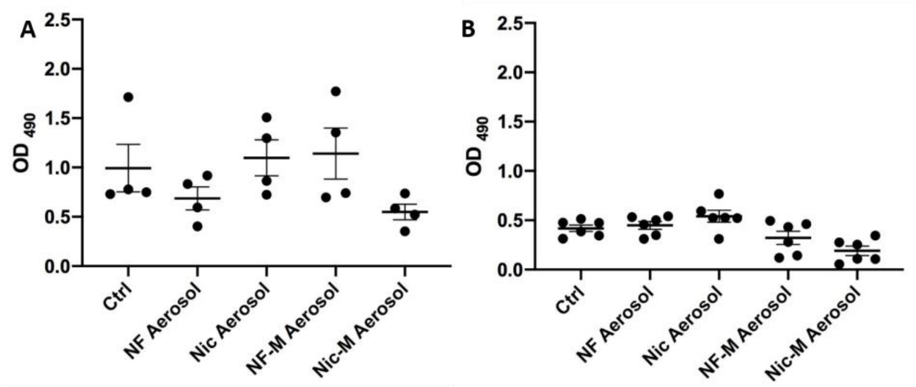

S4 Fig. Sucrose supplement did not affect biofilm formation upon e-cig exposure for any tested *Streptococcus* strains in this study.

Overnight cultures of *S. sanguinis* (A) and *S. gordonii* (B) were diluted 1:10 into e-cig pre-treated media with 1% sucrose supplementation and grown for 24 hours. Biofilms were stained with safranin and measured at OD_{490}. The data represent mean ± standard error of the mean (SEM) of one biological replicate out of 3. Groups were compared to control using one-way ANOVA (Dunnett's correction). $p<0.01$**; $p<0.001$***

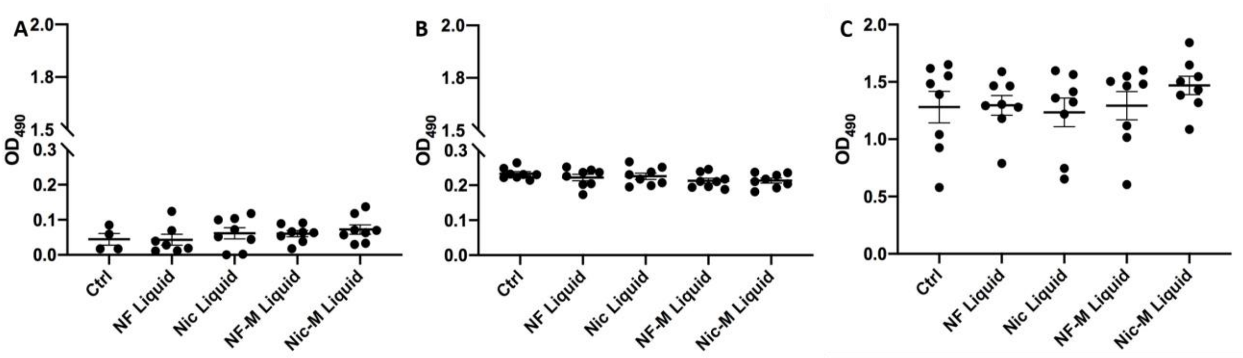

S5 Fig. E-liquid did not affect biofilm formation of any tested *Streptococcus* strains in this study.

For biofilm formation assays, overnight *S. sanguinis* (A) and *S. gordonii* (B), and *S. mutans* (C) cultures were diluted 1:10 into 1% e-liquid media and were grown 24 hours. Biofilms were stained with Safranin and measured at OD_{490}. The data represent mean ± standard error of the mean (SEM; n = 7) of one biological replicate out of 3.
